# *Glycine max* Fermented by a Novel Probiotic, *Bifidobacterium animalis* subsp. *lactis* LDTM 8102, Increases Immuno-Modulatory Function

**DOI:** 10.4014/jmb.2206.06038

**Published:** 2022-08-30

**Authors:** Jae Hwan Kim, Minju Jeong, Eun-Hee Doo, Young Tae Koo, Seon Joo Lee, Ji Won Jang, Jung Han Yoon Park, Chul Sung Huh, Sanguine Byun, Ki Won Lee

**Affiliations:** 1 Department of Biotechnology, Yonsei University, Seoul 03722, Republic of Korea; 2 Department of Agricultural Biotechnology, Seoul National University, Seoul 08826, Republic of Korea; 3 Department of Yuhan Biotechnology, School of Bio-Health Sciences, Yuhan University, Bucheon 14780, Republic of Korea; 4 Natural Products Convergence R&D Division, Kwangdong Pharm Co. Ltd., Republic of Korea; 5 Bio-MAX Institute, Seoul National University, 1 Gwanak-ro, Gwanak-gu, Seoul 08826, Republic of Korea; 6 Research Institute of Eco-friendly Livestock Science, Institute of Green-Bio Science and Technology, Seoul National University, Pyeongchang 25354, Republic of Korea; 7 Graduate School of International Agricultural Technology, Seoul National University, Pyeongchang 25354, Republic of Korea; 8 Advanced Institutes of Convergence Technology, Seoul National University, Suwon 16229, Republic of Korea; 9 Research Institute of Agriculture and Life Sciences, Seoul National University, Seoul 08826, Republic of Korea

**Keywords:** *Bifidobacterium animalis*, probiotics, immune stimulation, *Glycine max*, fermentation

## Abstract

Many probiotic species have been used as a fermentation starter for manufacturing functional food materials. We have isolated *Bifidobacterium animalis* subsp. *lactis* LDTM 8102 from the feces of infants as a novel strain for fermentation. While *Glycine max* has been known to display various bioactivities including anti-oxidant, anti-skin aging, and anti-cancer effects, the immune-modulatory effect of *Glycine max* has not been reported. In the current study, we have discovered that the extract of *Glycine max* fermented with *B. animalis* subsp. *lactis* LDTM 8102 (GFB 8102), could exert immuno-modulatory properties. GFB 8102 treatment increased the production of immune-stimulatory cytokines in RAW264.7 macrophages without any noticeable cytotoxicity. Analysis of the molecular mechanism revealed that GFB 8102 could upregulate MAPK2K and MAPK signaling pathways including ERK, p38, and JNK. GFB 8102 also increased the proliferation rate of splenocytes isolated from mice. In an animal study, administration of GFB 8102 partially recovered cyclophosphamide-mediated reduction in thymus and spleen weight. Moreover, splenocytes from the GFB 8102-treated group exhibited increased TNF-α, IL-6, and IL-1β production. Based on these findings, GFB 8102 could be a promising functional food material for enhancing immune function.

## Introduction

Recently, there has been growing interest in functional materials with immuno-modulatory activity [1]. According to many previous studies, it has reported that people with reduced immune responses are prone to immunodeficiency disorders [2]. People with low immunity are susceptible to infection from external pathogens and, as a result, can more easily suffer from various diseases [3]. Furthermore, this could lead to a variety of complications such as tuberculous meningitis, sepsis, pneumothorax, and cancer. Reduced immune system responses could also hinder recovery of wounds and autoimmune diseases [2]. Therefore, it is important to maintain a healthy immune system to prevent the onset of diseases caused by external and internal factors.

Among the many immuno-modulatory function materials, probiotics are one of the most studied materials. Reports on the various bioactivities of probiotics, such as defence against external pathogens [4], maintenance of gut health [5], and modulation of immunity [6, 7], have led to increase in the interest in and consumption of probiotics. Recently, probiotics have been used for the fermentation of food to enhance the bioactivities of these food materials [8, 9]. For example, ginseng fermented with probiotics exhibit superior properties to those fermented without probiotics. *Bifidobacterium* is a commercially popular probiotic that has been reported to be dominant in the guts of infants [10]. It has been demonstrated that foods fermented with *Bifidobacterium* exhibit diverse functions due to the enzymatic activities of the bacteria [11].

*Glycine max* is included in the legume species derived from East Asia and is widely known as soybean. In addition, *Glycine max* is a generally regarded as a safe food material as it has been consumed for a long time in East Asia. *Glycine max* has been reported to exhibit numerous healthy functions including anti-oxidant and anti-inflammatory effects in various chronic disease models [12-14]. It has been demonstrated that Asians who have consumed higher amounts of soybeans over a long period of time exhibited a lower risk of breast cancer compared to Western people who consumed smaller amounts of soybeans [15-17]. A fermented food made from soybeans is doenjang (soybean paste), which has been consumed for a long time in Korea. Deonjang is fermented by naturally occurring bacteria and fungi including *Bacillus subtilis*, *Aspergillus* species, *Rhizopus*, and *Mucor* [18]. Although doenjang has been reported to have various health-promoting properties, its molecular mechanism remains unclear [19-25].

In this study, novel *Bifidobacterium animalis* subsp. *lactis* LDTM 8102 was isolated from infants’ guts along with a wide range of *Bifidobacterium*. *Glycine max* was then fermented using only *B. animalis* subsp. *lactis* LDTM 8102 under controlled time and temperature. This is a novel preparation method for food fermented with *Glycine max*. It was hypothesized that the *Glycine max* fermented by *B. animalis* subsp. *lactis* LDTM 8102 (GFB 8102) in this study would possess higher bioactivity compared to *Glycine max* and *Glycine max* fermented with other types of commercial *Bifidobacterium*. Furthermore, the study attempted to identify the immuno-modulatory function of GFB 8102 in vivo and to discover its underlying molecular mechanism.

## Materials and Methods

### Isolation and Identification of *B. animalis* subsp. Lactis LDTM 8102 from Infant Feces

For this study, *B. animalis* subsp. *lactis* LDTM8102 [KCTC13392BP] with probiotic properties, such as higher acid and bile tolerance, antibiotic susceptibility, and β-glucosidase activity was isolated from healthy Korean infant feces. The experimental protocol was reviewed and approved by the Seoul National University Institutional Review Board (IRB NO. 1605/003-006). *Glycine max* powder used in the fermentation medium was obtained from Naturetech (Korea). *B. animalis* subsp. *lactis* LDTM8102 was cultivated twice in de Man, Rogosa, and Sharpe (MRS) broth supplemented with 0.05% L-cysteine-HCl (Sigma-Aldrich, USA) at 37°C for 24 h under anaerobic conditions as inoculum for fermentation. The *Glycine max* fermentation was anaerobically carried out at 37°C for 48 h at 70 ×*g* by inoculating the pre-culture at approximately 2 × 10^6^ CFU/ml into fresh 50 g/l *Glycine max* powder medium supplemented with 0.05% L-cysteine-HCl. The fermented *Glycine max* samples were heated at 95°C for 10 min to terminate microbial fermentation and stored overnight at -80°C before freeze-drying. The freeze-dried fermented *Glycine max* samples were kept at -80°C until analysis. Culture media, reagents, and materials were autoclaved at 121°C for 15 min prior to use.

### DNA Extraction and 16S ribosomal RNA (16S rRNA) Gene Sequencing

DNA was isolated from selected strain culture samples using the FastDNA SPIN Kit for Soil (MP Biomedicals, USA). The quality and quantity of DNA was determined using a SPECTROstar Nano microplate reader (BMG LABTECH GmbH, Germany). Bacterial identification was accomplished by 16S rRNA gene sequencing with the universal primers 27F (5’-AGA GTT TGA TCC TGG CTC AG-3’) and 1492R (5’-GGT TAC CTT GTT ACG ACT T-3’) (Macrogen Inc., Korea).

### Cell Culture

RAW264.7 murine macrophages (Korea Cell Line Bank, Korea) were incubated in Dulbeccós modified Eagle’s medium (DMEM) containing 10% fetal bovine serum (FBS) and 1% penicillin-streptomycin. The cells were cultured at 37°C under 5% CO_2_ conditions. RAW 264.7 macrophages (0.9 × 10^5^ cells) were seeded in 24-well plates and incubated for 24 h. Then, the cells were stimulated with 25 or 100 μg/ml of GFB 8102 for 6 h. The culture media was collected and centrifuged to abtain a supernant, which was used to measure the amount of pro-inflammatory cytokines, tumor necrosis factor (TNF)-α, and interleukin (IL)-6.

### Cytokine Analysis

Cytokine levels were measured according to the manufacturer's instructions, and enzyme-linked immunosorbent assay (ELISA) kit (R&D Systems, USA) was used. Briefly, coated capture antibody at 100 μl per well in a 96-well plate and incubate at room temperature overnight. After washing 3 times using washing buffer, 300 μl of reagent buffer was added per well for blocking, and incubated at room temperature for 1 h. Then, after washing 3 times using washing buffer, 100 μl of sample was added per well. After 2 h of incubation, the washing process is repeated. Then, added 100 μl of streptavidin-HRP per well and incubate for 20 min in a condition avoiding light. After repeating the washing process, add 100 μl of substrate solution per well. After incubation for 20 min, treat 20 μl of stop solution (2 N H_2_SO_4_) per well and incubate for 20 min. The absorbance value is measured by subtracting the value of the 540 nm wavelength from the 450 nm wavelength using the Varioskan Lux Multimode microplate reader (Thermo Fisher Scientific, USA).

### Cell Viability Assay

To identify cell viability, RAW264.7 macrophages were seeded into a 96-well white luminescence plate (SPL Life Science Co., Korea) at a density of 4 × 10^4^ cells/well and maintained in a 5% CO_2_ humidified chamber at 37°C for 24 h. The medium was then changed to serum-free DMEM and incubated for another 24 h. Afterward, various concentrations of GFB 8102 were applied to each well. After 24 h, the plates were treated with luminescence reagents for assessing cell viability according to the manufacturer’s instructions (Celltiter-glo Luminescent Cell Viability Assay Kit, Promega, USA). The plates were shaken for 1 min on an orbital shaker and incubated at room temperature for 10 min. Luminescence was analyzed using the Varioskan Lux Multimode microplate reader (Thermo Fisher Scientific).

### Endotoxin Testing

Endotoxin tests were conducted using Pierce LAL chromogenic endotoxin quantitation kits according to the manufacturer's protocols (Thermo Fisher Scientific). Briefly, preheat a 96-well plate at 37°C for 10 min. After 50 μl of standard solution and sample per well, 50 μl of LAL solution was added and incubated at 37°C for 10 min. After that, after processing 100 μl of chromogenic solution, shake gently and incubate for 6 min. Then, added 100 μl of stop solution (25% acetic acid) and measured the absorbance at a wavelength of 410 nm using the Varioskan Lux Multimode microplate reader (Thermo Fisher Scientific).

### Splenocyte Proliferation Assays

The experimental protocol was approved by the Animal Care and Use Committee of Seoul National University (SNU 170220-2-2). To isolate splenocytes, the spleen was removed from the mouse and washed with RPMI 1640 medium. After that, splenocytes were obtained by crushing the spleen and passing it through a 200 mesh stainless steel sieve. The splenocytes were washed with RPMI 1640 medium containing 10% FBS, and resuspended in ACK buffer (Gibco, USA) at 4°C for 3 min to remove red blood cells. The splenocytes were cultured in RPMI 1640 medium containing 10% FBS and seeded in 96-well plates treated with GFB 8102. After 2 days, the number of viable splenocytes were counted using Celltiter-Glo Luminescent cell viability assay kits according to the manufacturer's instructions (Promega).

### Immunoblot Analysis

RAW264.7 macrophages (2.25 × 10^6^ cells/ml) were treated with GFB 8102 for 1 h. The cells were then washed, scraped off the bottoms, and acquired in RIPA lysis buffer including protease and phosphatase inhibitors (Sigma–Aldrich, USA). The lysate was centrifuged to obtain a supernatant, which was then quantified using the Pierce BCA protein assay kit (Thermo Fisher Scientific). Proteins were separated using 10% SDS-PAGE and transferred to a nitrocellulose membrane (Bio-Rad, USA). After shaking in 5% skim milk in TBST (TBS containing 0.1%Tween 20) for 1 h to block the nitrocellulose membrane, the membrane was shaken overnight at 4°C with the primary antibody. After the membrane was washed with TBST, the membrane was incubated with the secondary antibody for 1 h. Then, after washing again with TBST, visualization was performed using westernlightning plus-ECL reagent (Cell Signaling Technology, USA). The following primary antibodies were used: phospho-p38 MAPK (cat. no. 4511; Cell Signaling Technology, Inc), p38 alpha/beta MAPK (cat. no. sc-7972; Santa Cruz Biotechnology, Inc.), phospho-SAPK/JNK (cat. no. 9251; cell signaling technology, Inc), JNK (cat. no. 9258; cell signaling technology, Inc), phospho-p44/42 MAPK (Erk1/2) (cat. no. 4370; Cell Signaling Technology, Inc), p44/ 42 MAPK (Erk1/2) (cat. no. 4695; cell signaling technology, Inc), phospho-MEK1/2 (cat. no. 9121; cell signaling technology, Inc), MEK1/2 (cat. no. 9122; cell signaling technology, Inc), phospho-MKK3/MKK6 (cat. no. 12280; Cell Signaling Technology, Inc), MKK3 (cat. no. 9138; Cell Signaling Technology, Inc).

### Animals

Male 6-week-old Balb/c mice were obtained from of Southeast Medi-Chem Institute (Korea). All experimental protocols were approved by the Institutional Animal Care and Use Committee (SEMI-20-002) of Southeast Medi-Chem Institute, Busan, Korea. The mice were treated with oral GFB 8102 for 19 days. Cyclophosphamide (Sigma-Aldrich) was injected into the mice at Days 17, 18, and 19 to induce immunosuppression. After sacrifice, organ weights were measured and spleens were isolated for cytokine analysis. Isolated spleens were washed with Roswell Park Memorial Institute (RPMI) 1640 medium, crushed to isolate the splenocytes, and passed through a 200-mesh stainless steel sieve to obtain a homogeneous cell suspension. The spleen suspension was washed twice with RPMI 1640-FBS medium, and the recovered splenocytes were resuspended in ACK buffer (Gibco) for 3 min to remove erythrocytes. The splenocytes were finally resuspended in 10% FBS RPMI 1640 and cultured in 96-well plates treated with lipopolysaccharides (LPS, Sigma-Aldrich). After 48 h, the supernatants were collected for cytokine analysis.

### Statistical Analysis

Graphs are presented as means of technical replicates with error range indicated. Software used was GraphPad Prism v5. Experimental data are expressed as mean ± standard deviation (SD). Animal studies are expressed as mean ± standard error of the mean (SEM) of three independent experiments performed for each experiment. One-way ANOVA followed by Duncan's test was used to compare significant differences among multiple groups. Two-tailed unpaired Student’s t test was used to compare between groups. A *p*-value <0.05 was used to indicate statistical significance.

## Results

### GFB 8102 Showed Increased Cytokine Production in RAW264.7 Macrophages

In order to analyze the function of GFB 8102, cytokine analysis was conducted in RAW264.7 macrophages since *Glycine max* has not been reported to exhibit immune-modulatory functions.

**Fig. 1:**
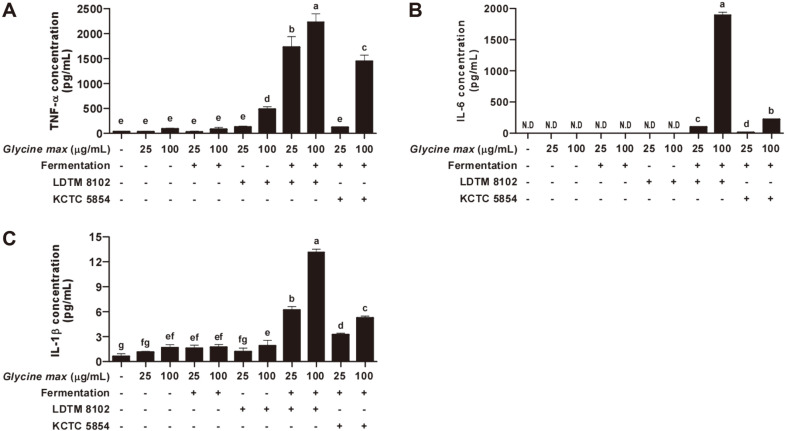
Cytokine production analysis after GFB 8102 treatment in RAW264.7 macrophages. (A-C) RAW264.7 macrophages were treated with GFB 8102 at the indicated concentrations, and the media was collected after 6 hours for TNF-α, IL-6, and IL-1β analysis. Data are presented as mean ± SD values of three independent experiments. *p < 0.05 represents a significant difference between the GFB 8102-treated group and GFB 5854-treated group (*n* = 3).

**Fig. 2:**
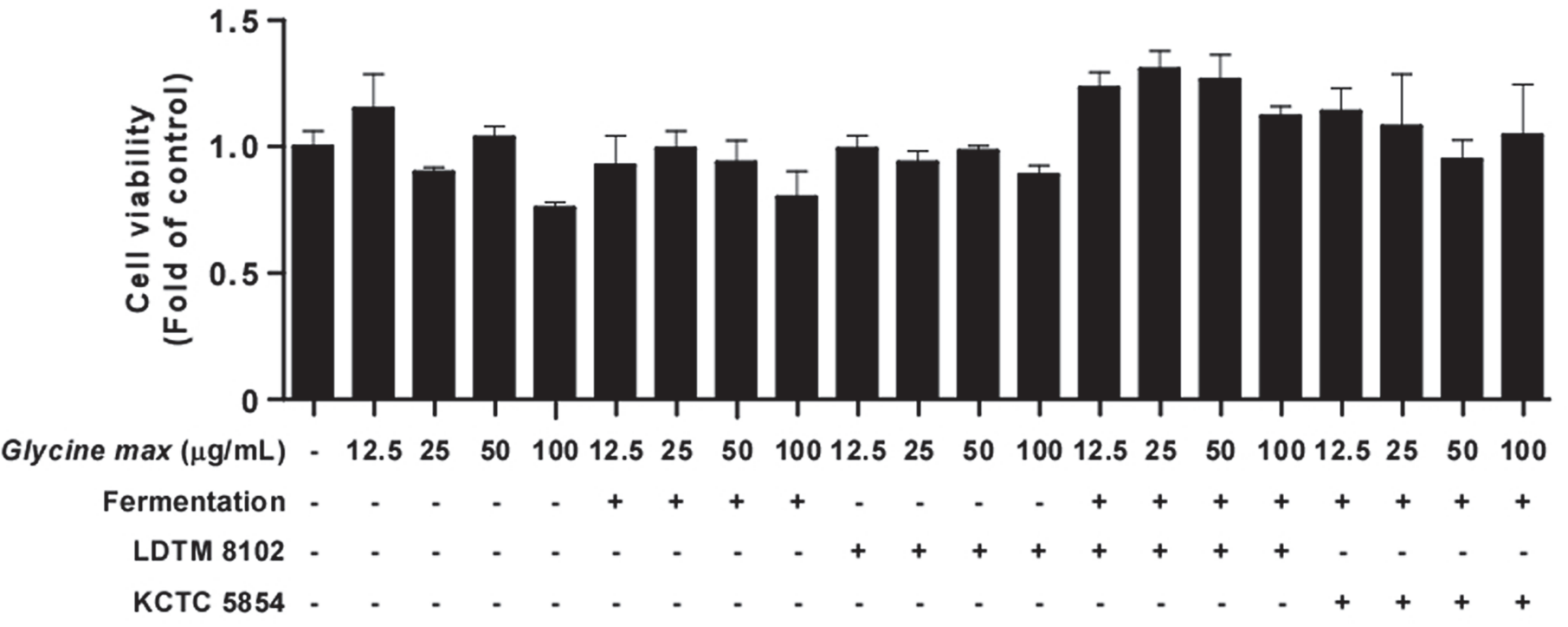
The effect of GFB 8102 on cell viability in RAW264.7 macrophages. RAW264.7 macrophages were treated with each sample at the indicated concentrations for 24 h. Data are presented as mean ± SD values of three independent experiments.

Macrophages are important cells in the human body as they act as inflammatory mediators controlling homeostasis and host defenses. Macrophages are particularly crucial in the innate immune system for defending the human body from external factors [26]. To protect against hazardous factors or pathogens including viruses, bacteria, and apoptotic cells, macrophages are activated and produce various cytokines such as tumor necrosis factor-α (TNF-α), interleukin-6 (IL-6), and interleukin-1β (IL-1β) [27, 28]. Therefore, the effects of GFB 8102 on cytokine production were analyzed in RAW264.7 macrophages (Fig. 1). Non-fermented *Glycine max* and *Glycine max* fermented without probiotics did not exhibit significant differences compared to the control group. Non-fermented *Glycine max* added to *B. animalis* subsp. *lactis* LDTM 8102 exhibited an increasing trend in TNF-α production but did not affect IL-6 and IL-1β production in RAW264.7 macrophages. However, upregulation of TNF-α, IL-6, and IL-1β production was observed after treatment with GFB 8102. Furthermore, the increased cytokine level of GFB 8102 was higher than the level of *Glycine max* fermented by *B. animalis* subsp. *lactis* KCTC 5854 (GFB 5854, commercial probiotics) at the same concentration (Figs. 1A-1C). In addition, GFB 8102 did not exhibit any cytotoxicity (Fig. 2) or endotoxicity (Table 1) in RAW264.7 macrophages. These results demonstrate that GFB 8102 can affect the activation of macrophages.

### Effect of GFB 8102 on Splenocyte Proliferation

**Fig. 3:**
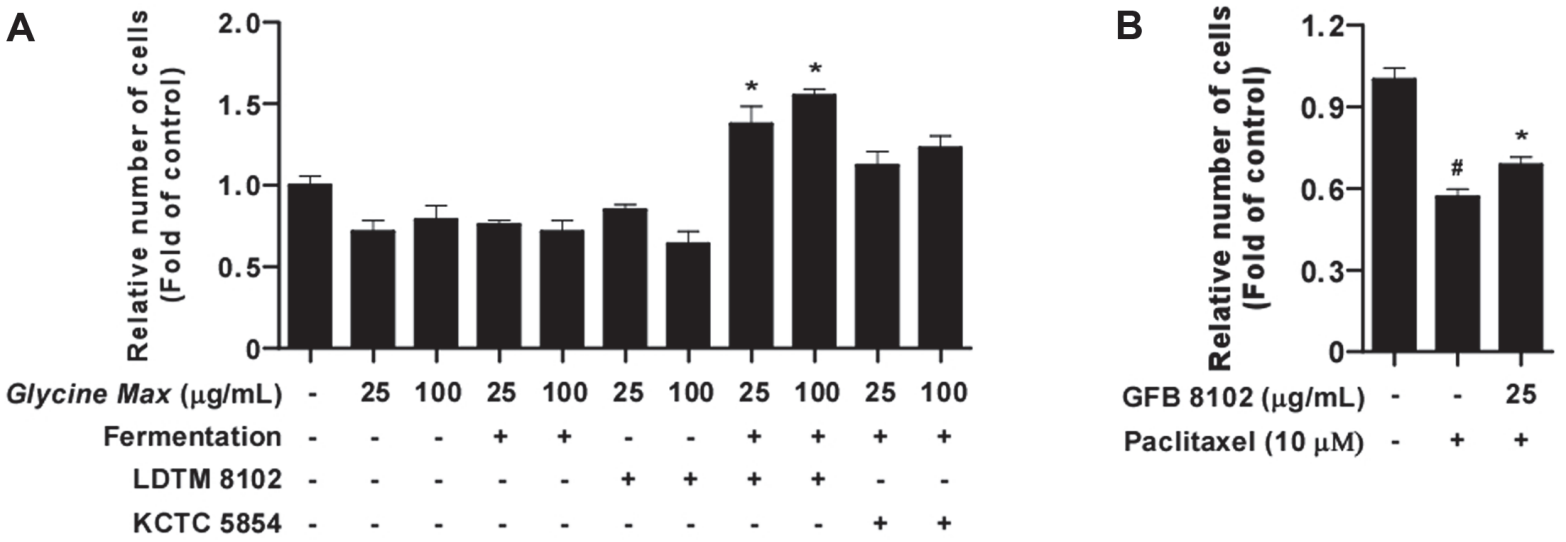
The effect of GFB 8102 on splenocyte proliferation. (**A**) Splenocyte proliferation was analyzed using the celltiter Glo assay. *p < 0.05 represents a significant difference between the GFB 8102-treated group and GFB 5854-treated group (*n* = 3). (**B**) Paclitaxel was used as a suppressor of splenocyte proliferation. #*p* < 0.05 represents significant difference between the control group and paclitaxel-treated group (*n* = 3). *p < 0.05 represents significant difference between the paclitaxel-treated group and GFB 8102-treated group (*n* = 3).

To identify whether GFB 8102 can affect primary immune cells, primary splenocytes were isolated from C57BL/6 mice. Increase of splenocyte proliferation represents activation of the immune system [29]. GFB 8102 significantly increased splenocyte proliferation compared to the control group. Moreover, GFB 8102 exhibited higher splenocyte proliferation compared to GFB 5854 (Fig. 3A). Paclitaxel, a cancer chemotherapeutic, has been reported to possess side effects including immunosuppression [30]. The paclitaxel-treated group demonstrated a lower proliferation rate compared to the control group. However, GFB 8102 significantly increased splenocyte proliferation suppressed by paclitaxel treatment (Fig. 3B). These findings demonstrate an effect of GFB 8102 on primary splenocyte proliferation ex vivo.

### GFB 8102 Targets the MAPK Pathway in RAW264.7 Macrophages

**Fig. 4:**
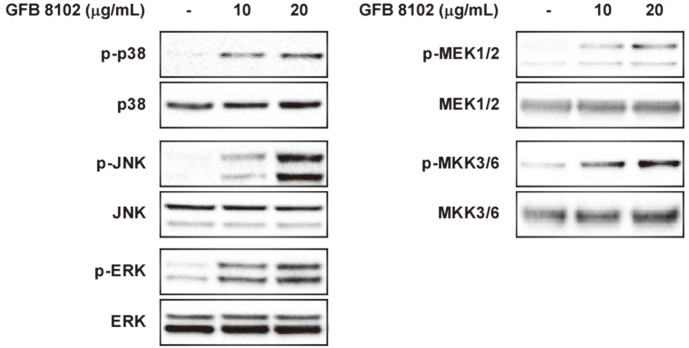
Involvement of the MAPK2 and MAPK signaling pathways in the immuno-modulatory function of GFB 8102. GFB 8102 was applied to RAW264.7 macrophages at a concentration of 10 or 20 μg/ml for 1 h.

To elucidate the molecular mechanism of GFB 8102 for activation of macrophages, the MAPK pathway was analyzed including ERK, p38, and JNK, which have been reported to be involved in macrophage activation [31]. In RAW264.7 macrophages, ERK, p38, and JNK signal transduction was upregulated when GFB 8102 was applied (Fig. 4). It was also shown that GFB 8102 regulates upsteam processes of MAPK. As a result, GFB 8102 has the potential to activate the MAPK2K-MAPK signaling pathway for stimulating immune responses.

### GFB 8102 Exhibits Immuno-Modulatory Function in vivo

**Fig. 5:**
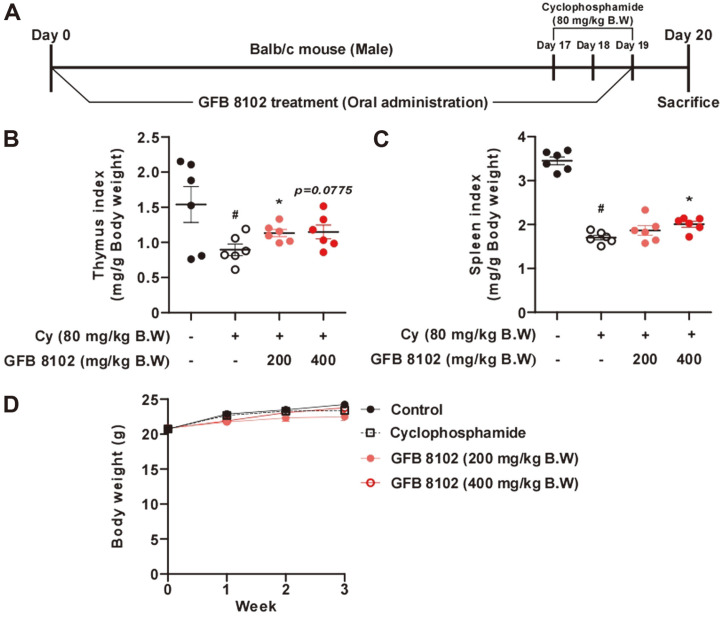
The immuno-modulatory effects of oral administration of GFB 8102 to Balb/C mice. (**A**) The schematic of the animal study. GFB 8102 were orally administrated to the mice for 19 days. (**B, C**) The weights of the organs were measured after sacrifice at Day 20. (**D**) The body weight changes of all groups were measured over the 19 days. #*p* < 0.05 represents a significant difference between the control group and cyclophosphamide (Cy)-treated group. *p < 0.05 represents a significant difference between the GFB 8102-treated group and cyclophosphamide-treated group. Data are presented as mean ± SEM values.

The potential of GFB 8102 in modulating immune responses in vivo was considered next. The animal experiment design is depicted in Fig. 5A. During oral administration of GFB 8102, cyclophosphamide was administered to the mice 3 times by intraperitoneal injection at 80 mg/kg body weight (Days 17, 18, and 19). The weights of the thymus and spleen have been known as biomarkers for immune responses in vivo [32]. After oral administration of GFB 8102 for 19 days, the organ weights of the thymus and spleen were analyzed. As expected, the cyclophosphamide-treated group exhibited significantly lower thymus and spleen weights compared to the control group. Interestingly, GFB 8102 significantly hindered the weight loss of the thymus and spleen caused by treatment with cyclophosphamide (Fig. 5B).

**Fig. 6:**
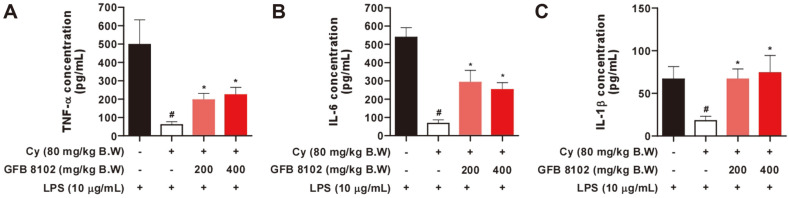
The cytokine production analysis of GFB 8102 in splenocytes. (**A-C**) Mice were treated with GFB 8102 at the indicated concentrations for 19 days. After isolation of splenocytes, LPS was administered at a concentration of 10 μg/ml. The supernatants were collected after 48 h for TNF-α, IL-6, and IL-1β analysis. Data are presented as mean ± SEM values of three independent experiments. #*p* < 0.05 represents a significant difference between the control group and cyclophosphamidetreated group. *p < 0.05 represents a significant difference between the GFB 8102-treated group and cyclophosphamide-treated group. Data are presented as mean ± SEM values.

It was then considered whether GFB 8102 could modulate cytokine production induced by lipopolysaccharides (LPS) in splenocytes. The cyclophosphamide-treated group exhibited significantly lower cytokine production including that of TNF-α, IL-6, and IL-1β compared to the control group (Fig. 6). However, the GFB 8102-treated group showed improvement in levels of TNF-α, IL-6, and IL-1β cytokine that were reduced by cyclophosphamide treatment. These results indicate that GFB 8102 possesses immuno-modulatory functions in vitro as well as in vivo.

## Discussion

Fermented foods originating from legume species are widely consumed in various Asian regions, and many studies have been performed to assess their health-promoting functions. In Korea, doenjang is a representative fermented soybean food that, which combined with *Glycine max* that has no known immuno-modulatory effects of itself exhibits immune-modulatory effects [33, 34]. However, the mode of action has yet to be discovered. Therefore, this study determined the enhanced immune functions of GFB 8102 including the molecular mechanisms of action in vitro and in vivo.

In this study, GFB 8102 was fermented using only one *Bifidobacterium* species under controlled processes. The production levels of TNF-α, IL-6, and IL-1β, which are involved in macrophage activation, were then analyzed. As expected, GFB 8102 exhibited higher cytokine levels in RAW264.7 macrophages compared to those of *Glycine max* fermented by *B. animalis* subsp. *lactis* LDTM 8102. *Glycine max* in the heat-killed *B. animalis* subsp. *lactis* LDTM 8102-treated group without fermentation exhibited higher TNF-α production in RAW264.7 macrophages compared to the control group. Thus, heat-killed *B. animalis* subsp. *lactis* LDTM 8102 has the potential to regulate cytokine production in macrophages, but changes of metabolites of *Glycine max* due to fermentation with *B. animalis* subsp. *lactis* LDTM 8102 are expected to be greater. In order to identify whether GFB 8102 can affect other immune cells, IL-2 production in Jurkat and NK-92 cells was analyzed. However, GFB 8102 did not demonstrate increased cytokine production. The macrophage stimulatory effects of GFB 8102 were involved in the MAPK signaling pathway. To assess the effects of GFB 8102 on splenocyte proliferation, isolated mouse splenocytes were used. Similar to cytokine production in macrophages, GFB 8102 exhibited higher splenocyte proliferation compared to the control group and GFB 5854-treated group. In addition, GFB 8102 has the potential to reduce side effects of paclitaxel including immune suppression through the up-regulatory effects of splenocyte proliferation.

In order to study the immune-modulatory effects of GFB 8102 in vivo, the immunosuppression model was tested with cyclophosphamide, which has been widely used for immunosuppression in animal models [35]. The weights of the thymus and spleen, which are composed of immune cells, increased in the GFB 8102-treated group compared to the cyclophosphamide-treated group. In addition, cytokines TNF-α, IL-6, and IL-1β induced by LPS for immune cell activation in splenocytes were analysed [36]. GFB 8102 demonstrated improvements in cytokine levels inhibited by cyclophosphamide.

This study revealed for the first time that *Glycine max* fermented by a novel probiotic, *B. animalis* subsp. *lactis* LDTM 8102, could influence the immune response through regulation of the MAPK signaling pathway. In addition, GFB 8102 could improve the reduced weights of the thymus and spleen and control cytokine production in cyclophosphamide-treated mouse models. These results demonstrate that GFB 8102 could be utilized as a potent immune-modulatory agent for use in functional food materials.

## Supplemental Materials

Supplementary data for this paper are available on-line only at http://jmb.or.kr.

## Figures and Tables

**Table 1:** Limulus Amebocyte Lysate (LAL) test of GFB 8102 for endotoxin testing.

Standard Endotoxin concentration (EU/ml)	Absorbance (405 nm)
100	0.140
10	0.128
1	0.121
0.1	0.058
0.01	0.051
0	0.050
