# Supplementary material for: Glycine max Fermented by a Novel Probiotic, Bifidobacterium animalis subsp. lactis LDTM 8102, Increases Immuno-Modulatory Function
Source: J Microbiol Biotechnol. 2022 Aug 30;32(9):1146–53. doi: 10.4014/jmb.2206.06038 (PMC9628972; doi:10.4014/jmb.2206.06038)
Supplement: Supplementary file 1 [file jmb-32-9-1146-supple.pdf]

J. Microbiol. Biotechnol.
https://doi.org/10.4014/jmb.2206.06038

Supplementary Table S1. The 16S ribosomal RNA gene sequence of *B. animalis* subsp. *lactis* LDTM8102 [KCTC13392BP]

Primer	16S ribosomal RNA gene sequence of *B. animalis* subsp. *lactis* LDTM8102
785F	TGGGGGTCGTGGATGCTGGATGTGGGGCCCTTTCCACGGGTCCCGTGTCG<br>GAGCCAACGCGTTAAGCATCCCGCCTGGGGAGTACGGCCGCAAGGCTAAA<br>ACTCAAAGAAATTGACGGGGGCCCGCACAAGCGGCGGAGCATGCGGATTA<br>ATTCGATGCAACGCGAAGAACCTTACCTGGGCTTGACATGTGCCGGATCG<br>CCGTGGAGACACGGTTTCCCTTCGGGGCCGGTTCACAGGTGGTGCATGGT<br>CGTCGTCAGCTCGTGTCGTGAGATGTTGGGTTAAGTCCCGCAACGAGCGC<br>AACCCTCGCCGCATGTTGCCAGCGGGTGATGCCGGGAACTCATGTGGGAC<br>CGCCGGGGTCAACTCGGAGGAAGGTGGGGATGACGTCAGATCATCATGCC<br>CCTTACGTCCAGGGCTTCACGCATGCTACAATGGCCGGTACAACGCGGTG<br>CGACACGGTGACGTGGGGCGGATCGCTGAAAACCGGTCTCAGTTCGGATC<br>GCAGTCTGCAACTCGACTGCGTGAAGGCGGAGTCGCTAGTAATCGCGGAT<br>CAGCAACGCCGCGGTGAATGCGTTCCCGGGCCTTGTACACACCGCCCGTC<br>AAGTCATGAAAGTGGGTAGCACCCGAAGCCGGTGGCCCGACCCTTGTGGG<br>GGGAGCCGTCTAAGGTGAGACTCGTGATTGGGACTAAGTCGTAACAGGTT<br>AACCCGTAAAATGGTGAGCCCCTTAAATTTTTAATCCCTAGGGGTAAGGA<br>CAAGGCCCCAAAACCCTCCCTTCCCCCTTGGGGTTCCTCCCCAAATTTCA<br>ACATTTCCCCCCTTTTCCCCGGGAATTTCCGTCTCCCCCCTCGAGCCTAT<br>CCCACCCCCCCCTTATCCGGGGCTGAAACCCCCCCTTTGAAGAGCGGAGA<br>TTTTTCCCCCCGAGCACAGCGAAAAACCCCAGCCAAAACCCTTTTTACCC<br>CCAAAAATCCACGAGGAAAAACCCCCCCCCCCCCCTAGTTTTTTAATCTT<br>GCGGGCTGGGACCCGACAAATAATCCCCATCCTTTTTTTTTTGAAAATCC<br>CCCCTCCTCTTGCAGGCCGAAGAAAGATTACTTTTTTCCTTTTTACAAAA<br>AAACGATTTTTTTTTATCCCCTAAGGACCACCCTTCCCTCCCCACCCCGA<br>CCGTTCGTTGGTTTTTGGTCCGGTTCTAGCCCCGTGCCTGGCCCAGGTAT<br>AATCCCCACCTCCGTGGCTCCCCCCCATCAAGGAAAACTATGGGGGGGTT<br>TTGGGCATCAAGCCCAACCTGTAGGTCTCTAATGGTTCTTCCTCCCCCCC<br>CCACCTTTCAACTACGGCGTCGCCCCCCCCCCCCCTATTTAAGGGAATGG<br>GGCGGGCCCGGTCCCCCCCCCCCCCCCCCCCCCTGTGAAACAAAAATGC<br>GGGTCCCATTCCCGGACCCCCCCCCCCATCCCCCCCCCCTTCACCCCCACTC

	ACTCCTGGCGGTCCCCCCCCCCCCTCCCGACCCACCCACCCCGAACATC<br>CGGACGGCCCCCCAACCCCAAGCCCCACCCCCTCCGCGCCC
907R	CCCAGTGGGCGTATCCCAGGCGGGATGCTTAACGCGTTGGCTCCGACACG<br>GGACCCGTGGAAAGGGCCCCACATCCAGCATCCACCGTTTACGGCGTGGA<br>CTACCAGGGTATCTAATCCTGTTCGCTCCCCACGCTTTCGCTCCTCAGCG<br>TCAGTGACGGCCCAGAGACCTGCCTTCGCCATTGGTGTTCTTCCCGATAT<br>CTACACATTCCACCGTTACACCGGGAATTCCAGTCTCCCCTACCGCACTC<br>CAGCCCGCCCGTACCCGGCGCAGATCCACCGTTAGGCGATGGACTTTCAC<br>ACCGGACGCGACGAACCGCCTACGAGCCCTTTACGCCCAATAAATCCGGA<br>TAACGCTCGCACCCTACGTATTACCGCGGCTGCTGGCACGTAGTTAGCCG<br>GTGCTTATTCGAACAATCCACTCAACACGGCCGAAACCGTGCCTTGCCCT<br>TGAACAAAAGCGGTTTACAACCCGAAGGCCTCCATCCCGCACGCGGCGTC<br>GCTGCATCAGGCTTGCGCCCATTGTGCAATATTCCCCACTGCTGCCTCCC<br>GTAGGAGTCTGGGCCGTATCTCAGTCCCAATGTGGCCGGTCACCCTCTCA<br>GGCCGGCTACCCGTCAACGCCTTGGTGGGCCATCACCCCGCCAACAAGCT<br>GATAGGACGCGACCCCATCCCATGCCGCAAAAGCATTTCCCACCCCACCA<br>TGCGATGGAGCGGAGCATCCGGTATTACCACCCGTTTCCAGGAGCTATTC<br>CGGTGCACAGGGCAGGTTGGTCACGCATTACTCACCCGTTCGCCACTCTC<br>ACCCCGACAGCAAGCTGCCAGGGATCCCGTTCGACTTGCATGTGTTAAGC<br>ACGCCGCCAGCGTTCATCCTGAGCCTGATTCCAAACTCTACACGTGGGGG<br>GGGGTTACAAATTTTGCGAAGTAACGGAATCTAAGGCTCAAAACTACACC<br>CGGTTACACTCTTTTCCTGTCT
